# In Situ Evaluation of Macrophage Populations and Inflammasome Components in Cutaneous and Mucocutaneous Leishmaniasis

**DOI:** 10.1111/pim.70026

**Published:** 2025-09-14

**Authors:** Caroline de Heleno Chagas de Carvalho, Gabriela Venicia Araujo Flores, Carmen Maria Sandoval Pacheco, Vania Lucia da Ribeiro da Matta, Carolina de Esteves de Morais, Ricardo Romiti, Walter Belda Júnior, Márcia Dalastra Laurenti

**Affiliations:** ^1^ Departamento de Dermatologia Hospital das Clínicas, Faculdade de Medicina, Universidade de São Paulo São Paulo Brazil; ^2^ Laboratório de Patologia de Moléstias Infcciosas (LIM‐50), Departamento de Patologia, Faculdade de Medicina Universidade de São Paulo São Paulo Brazil; ^3^ Institute of Biosciences São Paulo State University (UNESP) São Vicente Brazil; ^4^ Institute for Advanced Studies of Ocean São Paulo State University (UNESP) São Vicente Brazil; ^5^ Departamento de Patologia, Faculdade de Medicina Universidade de São Paulo São Paulo Brazil

**Keywords:** cutaneous leishmaniasis, histopathology, immunohistochemistry, inflammasome, macrophages, mucocutaneous leishmaniasis

## Abstract

American tegumentary leishmaniasis (ATL) affects the skin and mucous membranes, with a spectrum shaped by Th1/Th2 responses. This study investigated inflammasome activation in correlation with macrophage subpopulations, tissue parasitism, and histological changes in cutaneous and mucocutaneous leishmaniasis. We assessed inflammasome activation, tissue parasitism, and macrophage populations by immunohistochemistry, correlating with histopathological alterations using 29 biopsies from cutaneous and mucocutaneous leishmaniasis. Cutaneous leishmaniasis showed higher parasite density and infected macrophages than mucocutaneous leishmaniasis skin and mucosal lesions (*p* < 0.05). CD68^+^ and CD163^+^ macrophages were more abundant in cutaneous leishmaniasis (*p* < 0.0001 and *p* < 0.05). Inflammasome markers IL‐1β^+^ and IL‐18^+^ were significantly higher in cutaneous leishmaniasis (*p* < 0.05). In cutaneous leishmaniasis, CD68^+^ macrophages correlated positively with inflammasome markers, whereas in mucocutaneous leishmaniasis, CD163^+^ cells showed strong negative correlations with IL‐1β and caspase‐1. Parasite density correlated positively with inflammasome activation in cutaneous leishmaniasis but negatively in mucocutaneous leishmaniasis. Findings suggest that inflammasome activation plays different roles in ATL. In cutaneous leishmaniasis, inflammasomes contribute to the inflammatory response and parasite clearance, while in mucocutaneous leishmaniasis, they are less relevant, possibly due to a more defined immune response with minimal parasitism.

## 1 Introduction

American tegumentary leishmaniasis (ATL) is an infectious disease caused by protozoa of the genus *Leishmania*. *Leishmania (Viannia) braziliensis* is the main etiological agent in Brazil and Latin America [1, 2, 3, 4, 5, 6, 7]. In Brazil, there has been a progressive decline in the number of reported cases, from 26,328 in 2001 to 12,910 in 2023. Despite this downward trend, Brazil accounted for approximately 37% of the 34,954 cases reported in the Americas in 2023 [8].

Clinical progression depends on Th1/Th2 imbalance, influenced by the host's immunogenetic profile and species‐specific antigens of *Leishmania* [7, 9, 10, 11, 12]. The Th1 response, characterised by the production of IFN‐γ and TNF‐α, activates M1 macrophages, which exhibit a pro‐inflammatory and leishmanicidal profile with oxygen and nitrite metabolites [12, 13, 14]. In contrast, the Th2 response, marked by the release of IL‐4, IL‐10, and IL‐13, promotes the polarisation of M2 macrophages, associated with anti‐inflammatory processes, tissue repair, and parasite persistence [12, 13, 14, 15].

Cutaneous leishmaniasis (CL) typically presents as a single ulcer at the site of the sandfly bite, appearing after the full establishment of the cellular immune response. This clinical form is associated with a predominantly Th1‐mediated, functionally balanced immune response that promotes macrophage activation, induces localised tissue damage, and contributes to parasite control [1, 2, 6, 7, 11, 12, 15, 16, 17]. In contrast, mucosal lesions in mucocutaneous leishmaniasis (MCL) are characterised by a more aggressive inflammatory process, beginning with vasodilation and congestion and progressing to ulceration, necrosis, and, in advanced stages, cartilage destruction and perforation of the nasal septum or palate [1, 7, 9, 16]. This condition is accompanied by a more intense Th1 polarisation compared to patients without mucosal involvement, with increased CD4+ T cell infiltration, elevated production of IFN‐γ and TNF‐α, and reduced IL‐10 expression and frequency of regulatory monocytes [12].

Inflammasome activation plays a crucial role in leishmaniasis [3, 18, 19, 20, 21]. Inflammasomes are protein complexes composed of a sensor, an adaptor, and an inflammatory caspase. Upon activation by pathogen‐associated and damage‐associated molecular patterns, they trigger the release of proinflammatory cytokines such as IL‐1β and IL‐18. However, their role in the pathogenesis of leishmaniasis remains controversial [18, 19]. NLRP3 is the most extensively characterised inflammasome, and its activation by *Leishmania* spp. in macrophages regulates caspase‐1 activation, promoting cytokine secretion, nitric oxide (NO) production, and host cell pyroptosis [3, 18, 19, 21]. However, evidence indicates that when innate immune mechanisms fail to efficiently eliminate the parasites, persistent inflammasome activation may lead to excessive IL‐1β production, resulting in exacerbated inflammation, tissue damage, and worsening of disease progression [18, 19, 22, 23].

Despite advances in understanding inflammasome activation in leishmaniasis, knowledge gaps persist. Therefore, this study aims to evaluate how M1/M2 macrophages, inflammasome components, and tissue parasitism interact in cutaneous and mucocutaneous leishmaniasis, addressing gaps in understanding their roles in the immunopathological mechanisms involved.

## 2 Material and Methods

### 2.1 Patients and Samples

This study included biopsy blocks from skin and mucosal samples of CL and MCL patients collected between 2011 and 2019. These samples were stored at the Dermatopathology Laboratory of the Department of Dermatology, Hospital das Clínicas, Faculty of Medicine, University of São Paulo (FMUSP).

The inclusion criteria were histopathological examination describing granulomatous dermatitis with plasma cells and a confirmed leishmaniasis diagnosis based on at least one of the following: a positive delayed hypersensitivity (DTH) reaction, a positive parasitological diagnosis by immunohistochemistry, and/or a positive molecular diagnosis by PCR.

### 2.2 Molecular Diagnosis and Species Identification

DNA extraction from paraffin‐embedded skin and mucosa biopsy sections from patients with CL and MCL was performed using the QIAamp DNA formalin‐fixed, paraffin‐embedded Tissue kit (QIAGEN, Germany). *Leishmania* genus detection was performed by PCR using Leish1: 5′‐AACTTTTCTCTGGTCCTCCGGGTAG‐3′ and Leish2: 5′‐ACCCCCAGTTTCCCGCC‐3′ primers to amplify a 120‐base pair (bp) product [24]. PCR‐restriction fragment length polymorphism (RFLP) was performed to characterise *Leishmania* species, which amplified a 234 bp fragment of the *hsp*70 gene (*hsp*70C sense: 5′GGACGAGATCGAGCGCATGGT′3 and antisense: 5′TCCTTC GACGCCTCCTGGTTG′3) [25]. The Internal Transcribed Spacer 1 (ITS1) PCR was used, amplifying a 300 to 350 bp (LITSR: 5′CTG GAT CAT TTT CCG ATG′3 and L5.8S: 5′TGA TAC CAC TTA TCG CAC TT′3) [26]. PCR products were digested with the *Hae*III enzyme (New England Biolabs, USA) and analysed on a 4% agarose gel by electrophoresis. DNA from reference strains of *Leishmania*, *Leishmania braziliensis* (MHOM/BR/1995/M15280), *Leishmania amazonensis* (MHOM/BR/1973/M2269), *L. shawi* (MCEB/BR/1984/M8408), and *Leishmania infantum* (MHOM/BR/1974/PP75) was included in the PCR‐RFLP reaction.

### 2.3 Histopathological Study

Haematoxylin–eosin (HE) stained histological sections were examined under an optical microscope to characterise the inflammatory response and tissue parasitism. The histopathological analysis, a semiquantitative comparative evaluation of the HE‐stained sections, was performed. The intensity of different processes was evaluated by two independent pathologists using a modified Ridley and Ridley method [27], as follows: (−) negative, 1–5 cells (+) rare, 5–50 cells (++) mild, 50–100 cells (+++) moderate, and > 100 cells (++++) intense.

### 2.4 Immunohistochemical Study

Immunohistochemistry was performed on paraffin‐embedded biopsy slides of skin and mucosal tissue to evaluate the involvement of macrophage subpopulations (M1: CD68 and NOS2; M2: CD163 and IL‐10) and inflammasome components (IL‐1β, IL‐18, and Caspase‐1) in the tissue inflammatory response. The immunohistochemical procedure was conducted according to Sandoval et al. [28] using as primary antibodies: mouse anti‐CD68 monoclonal (ab955, ABCAM) 1:400, anti‐CD163 monoclonal (ab156769, ABCAM) 1:400, rabbit anti‐NOS2 polyclonal (ab15323, ABCAM) 1:50, anti‐IL‐10 polyclonal (ab34843, ABCAM) 1:1000, anti‐IL‐1β polyclonal (ab2105, ABCAM) 1:300, anti‐IL‐18 polyclonal (ab68435, ABCAM) 1:1500, anti‐Caspase‐1 monoclonal (G6231‐3D2, Sigma‐Aldrich) 1:500, and mouse anti‐leishmania, hyperimmune serum (LIM‐50/HCFMUSP) 1:1000.

### 2.5 Quantitative Morphometric Analysis of Immunolabelled Cells

Immunohistochemically stained sections were analysed under an optical microscope using AxioVision 4.8. Ten fields per section (40× objective) were photographed, and brown‐stained cells were quantified with ImageJ. Cell density was calculated as the mean cell count divided by the image area.

### 2.6 Statistical Analysis

Statistical analysis was performed using GraphPad Prism 10. Normality was assessed by the Kolmogorov–Smirnov test. Group comparisons used *t*‐tests or Mann–Whitney tests as appropriate; correlations were evaluated by Pearson or Spearman tests based on data distribution. Significant differences were considered when *p*‐values were < 0.05.

## 3 Results

### 3.1 Characterisation of Sampling

Of the 29 tissue blocks analysed, 20 were from CL skin biopsies and nine from MCL lesions (six skin, three mucosal). In CL cases, 62% showed positive DTH, 85% were immunohistochemistry‐positive for amastigotes, and 95% tested PCR‐positive. In MCL cases, DTH was positive in 100%, immunohistochemistry in 67%, and PCR in 90%.

### 3.2 Histopathological Study

**FIGURE 1:**
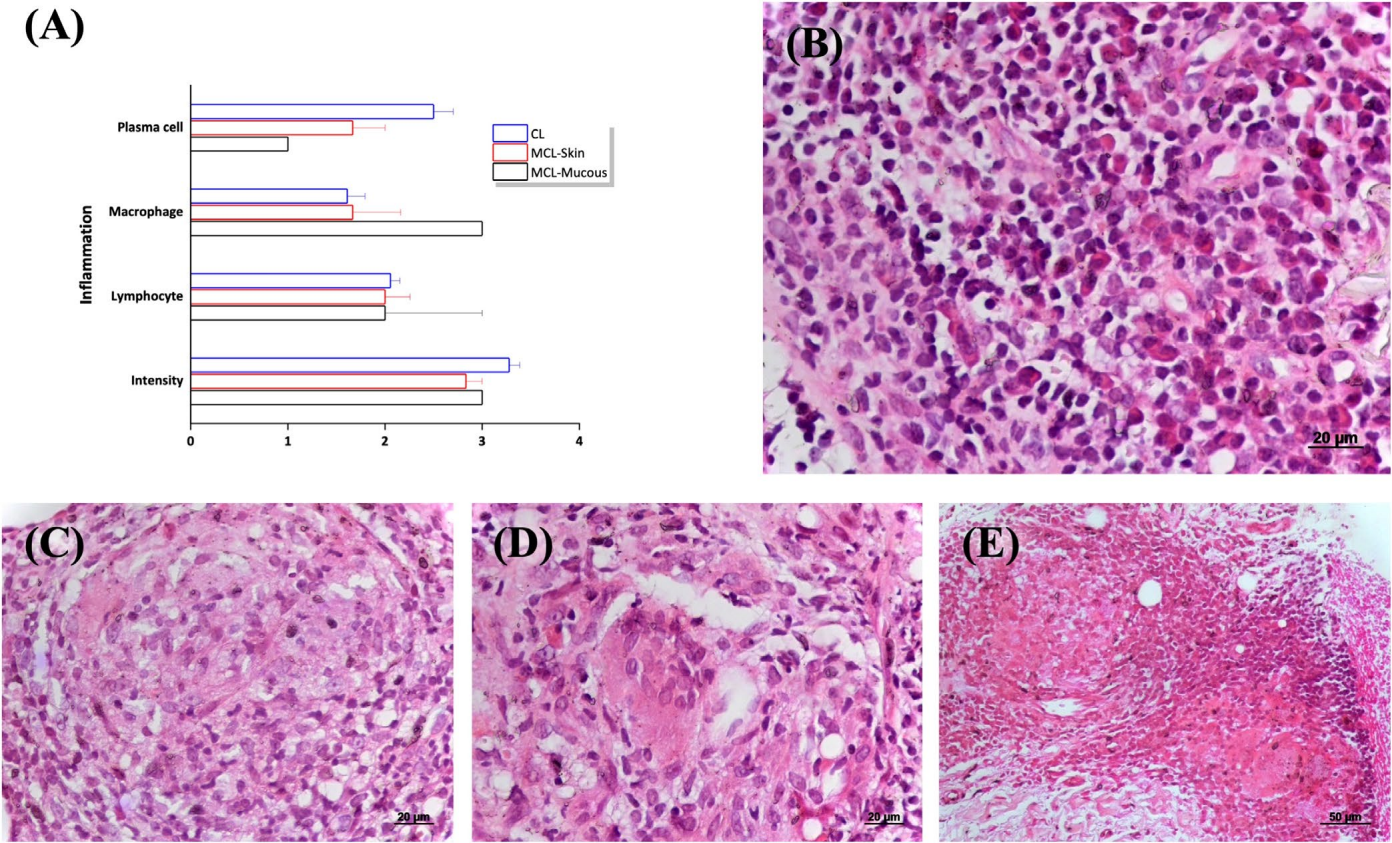
Graph showing the semiquantitative analysis of histopathological alterations observed in the dermis and mucosa (A) of histological sections from skin biopsy samples of patients affected by cutaneous leishmaniasis (CL) and mucocutaneous leishmaniasis (MCL). Illustrative image of paraffin histological section of skin lesions of patients exhibiting inflammatory infiltrate in the dermis (B), epithelioid granuloma (C), multinuclear giant cells (D), and focal areas of necrosis (E). Haematoxylin and eosin staining; magnification: A/B/C/D, 40×; E, 20×.

Exocytosis, thinning, and acanthosis were observed in the epithelial tissue, which was rare in MCL and both rare and mild in CL. Dermal inflammatory infiltration consisted of plasma cells, macrophages, and lymphocytes, with moderate to intense levels and no significant differences between groups, highlighting the degree of inflammation in the analysed samples (Figure 1A,B). Granulomatous formations, including early‐stage granulomas and well‐formed epithelioid granulomas, were observed in 50% of CL and 56% of MCL cases. Multinucleated giant cells were present in 50% of CL and 75% of MCL cases, while focal areas of necrosis were detected in 94% of CL and 64% of MCL cases (Figure 1C–E).

No structures suggestive of *Leishmania* amastigotes were identified in the histopathological analysis. However, immunohistochemical staining confirmed the presence of parasites in 85% of CL and 67% of MCL cases.

### 3.3 Identification of *Leishmania* Parasites

PCR‐kDNA detected *Leishmania* spp. in 93% (27/29) of paraffin‐embedded biopsies. Positive samples were analysed by PCR‐RFLP (Hsp70 and ITS1) with *Hae*III digestion, identifying species in nine cases: six as *Leishmania* (*V*.) *braziliensis* and three as *Leishmania* (*L*.) *amazonensis* (Supporting 1). *L. (V.) braziliensis* was identified in four skin lesions of CL, in one skin and one mucosal lesion of MCL; *L. (L.) amazonensis* was identified in three skin lesions of CL.

### 3.4 Immunohistochemistry Study

#### 3.4.1 Determination of Tissue Parasitic Load

**FIGURE 2:**
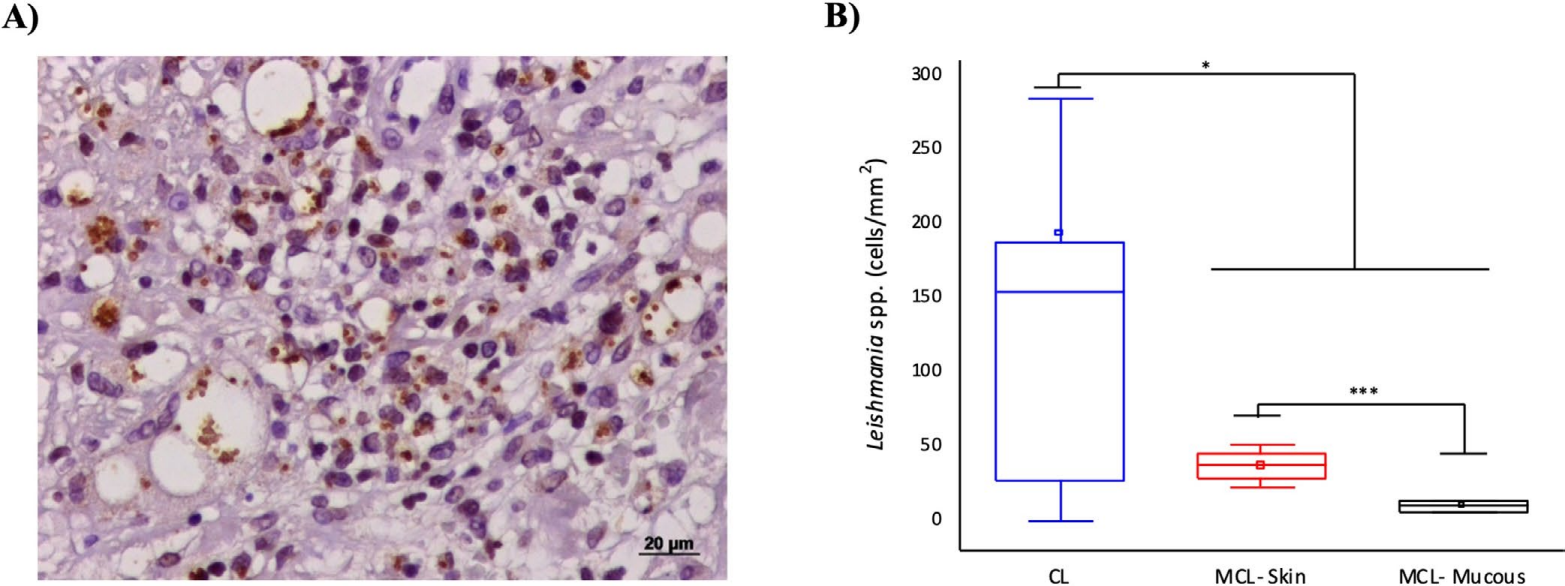
Representative image of the immunohistochemistry reaction performed on a histological section of a skin biopsy from patients with cutaneous leishmaniasis (CL), showing *Leishmania* amastigote forms in brown colour and infected macrophages (A). Box‐plot graph displaying the mean, median, maximum, and minimum values of *Leishmania* amastigote density per square millimetre in cutaneous leishmaniasis (CL) and mucocutaneous leishmaniasis—skin (MCL‐Skin) and mucosa (MCL‐Mucous) (B). Student's *t* test was used to compare each sample and * indicate *p* ≤ 0.05. DAB staining, objective 40×.

The average parasite density, assessed by immunohistochemistry in patients with CL, was 340.4 ± 155.2 parasites/mm^2^, whereas in skin lesions of patients with MCL it was 36.5 ± 6.1 and in mucosal tissue 9.5 ± 4.1. Parasite density was significantly higher in CL patients compared to the skin and mucosa of MCL patients. Among MCL patients, parasite density was greater in skin lesions than in mucosal lesions (*p* < 0.05) (Figure 2). Regarding the mean density of infected macrophages, the highest values were observed in CL patients (91.3 ± 44.7), followed by the skin of MCL patients (12.2 ± 7.0) and mucosal tissue (1.4 ± 1.4) (*p* < 0.05). Interestingly, when analysing the ratio between macrophage density with particulate and/or homogeneous antigenic material and the density of macrophages infected with *Leishmania* amastigotes, we found that this ratio was highest in mucosal lesions (9.6), followed by skin lesions (2.83) in MCL, and was lowest (0.74) in CL patients (Figure 2).

#### 3.4.2 Characterisation of Macrophage Subpopulations

**FIGURE 3:**
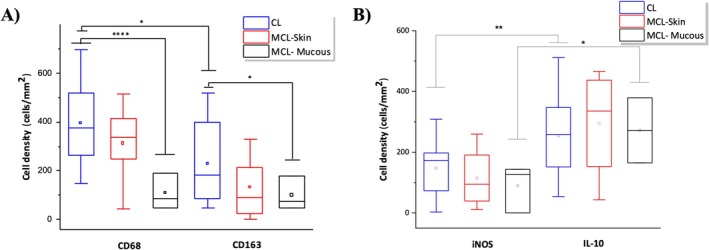
Box plot graph showing the mean, median, maximum, and minimum values of the density of CD68 and CD163 cells (A) and iNOS and IL‐10 cells (B) in cutaneous leishmaniasis (CL), mucocutaneous leishmaniasis—skin (MCL‐Skin), and mucosa (MCL‐Mucous). Student's *t* test was used to compare each sample and * indicates *p* < 0.05; ** indicates *p* < 0.01; **** indicates *p* < 0.0001.

The density of immunolabelled cells in the biopsies from patients was higher than observed in normal skin (*p* < 0.05). In normal skin, the density of CD68^+^ was 17.9 ± 8 while the density of CD163^+^ was 14.3 ± 6.8 cells/mm^2^. We observed a higher density of CD68^+^ cells in skin lesions of CL (397.2 ± 35.8) compared to mucosal lesions in MCL (108.2 ± 43.5) (*p* < 0.0001). Similarly, the density of CD163^+^ cells was higher in skin lesions of CL (234.5 ± 36.7 cells/mm^2^) compared to mucosal lesions of MCL (100.1 ± 40.2) (*p* < 0.05). The density of CD68^+^ cells (311.9 ± 80 cells/mm^2^) as well as CD163^+^ cells (131.5 ± 52) in the skin lesion of MCL did not show a statistical difference between the skin lesion of CL and the mucous lesion of MCL (*p* > 0.05). The density of CD68^+^ cells was significantly higher than that of CD163^+^ cells in skin lesions of CL (*p* < 0.05) (Figure 3A) (Supporting 2).

We observed a higher expression of IL‐10^+^ than iNOS^+^ cells in both skin CL biopsies and mucosal MCL biopsies (*p* < 0.05). The density of iNOS^+^ cells was 145.6 ± 22.1 cells/mm^2^ in skin lesions of CL, 115.0 ± 47.7 in the skin and 90.2 ± 45.3 in the mucosa of MCL. In contrast, the density of IL‐10^+^ cells was 274.1 ± 28.9 cells/mm^2^ in the skin of CL, 295.0 ± 84.2 in the skin and 272.0 ± 87.3 in the mucosa of MCL. Statistical differences were not observed among the groups (LC, MCL‐skin, MCL‐mucous), neither for iNOS+ nor for IL‐10^+^ cells (Figure 3B).

The density of immunolabelled iNOS^+^ and IL‐10^+^ cells in CL and MCL was higher than that observed in normal skin, where the density of iNOS^+^ cells was 0.1 ± 0.1 cells/mm^2^ and IL‐10^+^ cells was 13.3 ± 5.

In the absence of double staining, M1 (CD68+/iNOS+) and M2 (CD163+/IL‐10+) macrophages were inferred through correlation analysis between markers.

We observed a moderate positive correlation (*p* = 0.0463, *r*_s_ = 0.4892) between CD68^+^ and iNOS^+^ cells in CL, suggesting that a portion of CD68^+^ macrophages belongs to the M1 subtype. Similarly, we found a moderate positive correlation (*p* = 0.0165, *ρ* = 0.5421) between CD163^+^ and IL10^+^ cells, indicating that a subset of CD163^+^ cells is M2 macrophages in CL.

No correlation was observed between CD68^+^ and iNOS^+^ cells in skin or mucosal lesions of MCL, or between CD163^+^ and IL10^+^cells.

A moderate positive correlation was observed between parasite density and the density of IL‐10^+^ cells in CL (*p* = 0.021, *ρ* = 0.579). In contrast, in mucosal lesions of MCL, this correlation was strong and negative (*p* = 0.0001, *r*_s_ = −1.000). Additionally, a moderate positive correlation was observed between the density of macrophages containing antigenic material and the density of iNOS^+^ cells in CL (*p* = 0.0473, *r*_s_ = 0.5193). Similarly, a strong positive correlation was found between the density of macrophages containing antigenic material and the density of iNOS^+^ cells in mucosal lesions of MCL (*p* = 0.0027, *r*_s_ = 0.9828).

#### 3.4.3 Characterisation of Inflammasome Markers

**FIGURE 4:**
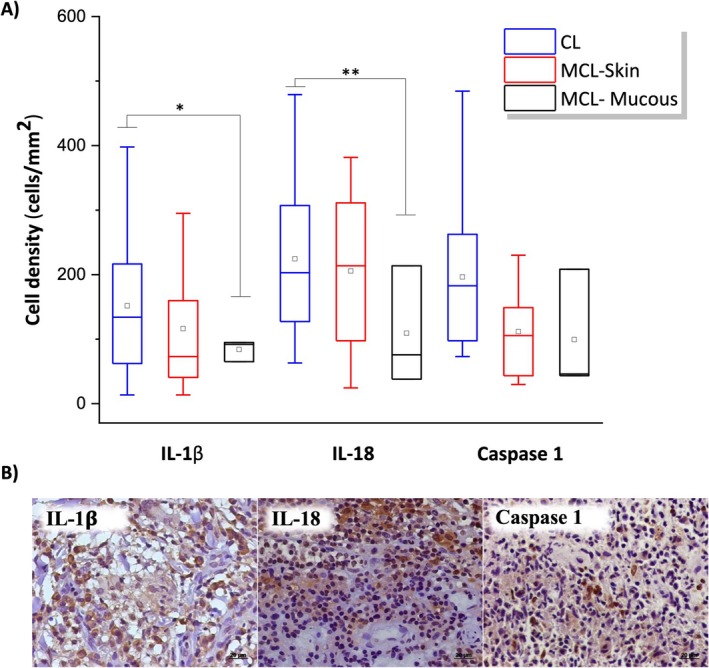
(A) Box plot graph showing the mean, median, maximum, and minimum values of the density of IL‐1β, IL‐18, and caspase‐1 cells in cutaneous leishmaniasis (CL), mucocutaneous leishmaniasis—skin (MCL‐Skin), and mucosa (MCL‐Mucous). (B) Representative immunohistochemical staining of a histological section from a skin biopsy of patients with cutaneous leishmaniasis, showing IL‐1β^+^, IL‐18^+,^ and caspase‐1^+^ cells. Student's *t* test was used to compare each sample and * indicates *p* < 0.05; ** indicates *p* < 0.01. DAB staining, objective 40×.

Inflammasome marker analysis revealed a higher density of immunolabelled cells in leishmaniasis cases than in normal skin. In normal skin, the density was 0.1 ± 0.1 for IL‐1β, 0.1 ± 0.1 for IL‐18, and 1.0 ± 1.0 cells/mm^2^ for caspase‐1. The density of IL‐1β^+^ and IL‐18^+^ cells was significantly higher in CL than in mucosal lesions of MCL (*p* < 0.05). Specifically, the densities of IL‐1β^+^, IL‐18^+^, and caspase‐1 cells were: 164.6 ± 24.7, 233.8 ± 26.3, and 207.9 ± 25.9 cells/mm^2^ in CL; 116.4 ± 51.0, 205.7 ± 65.9, and 111.5 ± 36.6 in skin lesions; and 83.9 ± 9.5, 109.1 ± 53.4, and 99.2 ± 54.6 in mucosal lesions of MCL, respectively (Figure 4).

A moderate positive correlation between CD68 and IL‐1β (*p* = 0.0042, *r*_s_ = 0.6115) and between CD68 and caspase‐1 (*p* = 0.0272, *r*_s_ = 0.4930), as well as a strong positive correlation between CD68 and IL‐18 (*p* < 0.0001, *r*_s_ = 0.8582), was observed in CL. Regarding CD163, a moderate positive correlation was observed with all inflammasome markers in CL (IL‐1β—*p* = 0.0134, *ρ* = 0.5564; IL‐18—*p* = 0.0046, *ρ* = 0.6207; Caspase 1—*p* = 0.0125, *ρ* = 0.5606). In MCL, a strong positive correlation was found between CD163 and IL‐18 in skin lesions (*p* = 0.0079, *r*_s_ = 0.9647). However, in mucosal lesions, a strong negative correlation was observed between CD68 and IL‐1β (*p* = 0.0018, *r*_s_ = −0.9377), CD163 and IL‐1β (*p* = < 0.0001, *r*_s_ = −0.9911), and CD163 and caspase‐1 (*p* = 0.0127, *ρ* = −0.8868).

Additionally, we observed a moderate positive correlation between parasite density (*p* = 0.0093, *ρ* = 0.6377), parasitized macrophages (*p* = 0.0215, *ρ* = 0.5372), macrophages containing antigenic material (*p* = 0.0282, *r*_s_ = 0.5166), and the density of IL‐1β^+^ cells in CL. Similarly, we found a moderate positive correlation between parasite density (*p* = 0.0360, *ρ* = 0.5324), macrophages containing antigenic material (*p* = 0.0144, *p* = 0.0144), and the density of IL‐18^+^ cells. Furthermore, a strong positive correlation was observed between parasitized macrophages (*p* < 0.0001, *ρ* = 0.8198) and the density of IL‐18^+^ cells in CL. In MCL, we observed a strong positive correlation between parasite density and IL‐18^+^ cell density (*p* = 0.0003, *r*_s_ = 0.9961). Moreover, a strong negative correlation was found between the density of parasitized macrophages and IL‐18^+^ cell density (*p* = 0.0001, *r*_s_ = −0.9976), as well as between macrophages containing antigenic material and IL‐18^+^ cell density (*p* = 0.0003, *r*_s_ = −0.9963).

## 4 Discussion

The role of inflammasome activation in ATL remains controversial. While NLRP3‐driven IL‐1β production can enhance parasite elimination via nitric oxide induction, excessive IL‐1β exacerbates inflammation and tissue damage when parasites persist [22]. *Leishmania* may inhibit NLRP3 activation as an immune evasion strategy, promoting parasite persistence and the development of chronic, long‐lasting disease [18]. Studies show contrasting outcomes depending on the parasite species; IL‐1β and caspase‐1 levels inversely correlate with parasite load in *L. (V.) panamensis* infection [19], whereas *L. (V.) guyanensis* appears to evade inflammasome activation altogether [29], suggesting species‐specific immunomodulation strategies.

Immunohistochemistry confirmed the presence of amastigote forms in 85% of CL and 67% of MCL cases, with higher parasite density in CL biopsies, followed by skin and mucosal samples from MCL. Infected macrophage density was also greater in CL, consistent with previous reports [30].

The ratio of macrophages containing antigenic material to the density of macrophages infected with amastigote forms was highest in mucosal biopsies, followed by skin MCL and CL biopsies. This likely reflects strong Th1‐driven CD4^+^ T cell responses promoting macrophage activation and parasite destruction [31]. Consistently, a positive DTH response was observed in 100% of MCL cases but only 62% of CL cases.

Although significant differences were found in parasite density and infected macrophages, the histopathological changes appeared similar across different clinical forms. Histopathological analysis suggests slight variability in the changes in the dermis and epidermis [32, 33].

We observed a 93% positive rate in the samples analysed and identified the species in nine cases. No statistically significant difference was found between infections caused by *L. (V.) braziliensis* and *L. (L.) amazonensis* regarding the markers used, possibly due to the limited sample size. In addition to the small sample size (*n* = 29), we used archival formalin‐fixed, paraffin‐embedded tissues, which may have led to DNA degradation and reduced PCR sensitivity, limiting definitive species parasite identification.

Macrophages play a key role in defending against parasitic infections, acting as host cells for the parasite during acute and chronic leishmaniasis [34]. They function as antiparasitic effectors and immunoregulators, with M1 (inflammatory) and M2 (anti‐inflammatory) subpopulations, where cytokines, microenvironmental factors, host metabolites, and parasitic products regulate their activation. M1 macrophages produce cytokines like IL‐6, IL‐12, and TNF to initiate the inflammatory response, while M2 macrophages support tissue repair and anti‐inflammatory functions through the Th2 response [13, 14].

To characterise macrophage subpopulations, we used antibodies targeting NOS2 and CD68 for M1 macrophages and IL‐10 and CD163 for M2 macrophages. M1 and M2 cell densities were higher in CL skin lesions than in mucosal lesions in MCL, indicating a predominant involvement of different macrophage subpopulations in CL.

M1 macrophages play an important role in activating the cellular immune response to eliminate the parasite. Conversely, M2 macrophages target the immune system to modulate inflammation and prevent excessive tissue damage [35, 36]. We observed a higher IL‐10^+^ than NOS2^+^ cell density, with IL‐10^+^ cells correlating positively with parasite burden in CL and negatively in mucosal lesions. Besides M2 macrophages, lymphocyte subpopulations are significant IL‐10 sources in leishmaniasis [37, 38].

In CL skin lesions, Th2‐type CD4^+^ T lymphocytes and M2 macrophages may contribute to IL‐10 production, within a mixed cellular immune response [39]. In contrast, Tregs appear to be the main IL‐10 source in MCL mucosal lesions, modulating inflammation to limit tissue damage [40]. Consistently, we observed a negative correlation between parasite density and IL‐10^+^ cells in mucosa, and a strong positive correlation between macrophages containing antigenic material and iNOS^+^ cells, indicating macrophage activation for NO‐mediated parasite control [41].

The NLRP3 inflammasome is the best‐characterised and plays a key role in controlling *Leishmania* replication in macrophages [18, 19]. Its activation requires two steps: first, microbial components or TNF‐α activate toll‐like receptors (TLRs), inducing the expression of inflammatory genes like NLRP3 and IL‐1β. Second, microbial toxins cause membrane damage, potassium efflux, ROS production, and lysosomal cathepsin release, triggering canonical NLRP3 activation. This leads to Caspase‐1 activation, promoting the release of IL‐1β and IL‐18 and triggering pyroptosis [18, 20, 21].

We observed the presence of all inflammasome markers (IL‐1β, IL‐18, and Caspase‐1) in both CL and MCL. IL‐1β and IL‐18‐positive cells were higher in CL compared to mucosal lesions in MCL, suggesting a more pronounced involvement of these elements in the inflammatory process in CL. Supporting our findings, a study combining PCR array, qPCR, and immunohistochemical analysis identified upregulated inflammasome‐related genes, such as IL‐1β and NLRP3, in CL patients. Notably, in CL caused by *L. (V.) braziliensis*, NLRP3 expression increased during early stages and declined in chronic phases, suggesting distinct roles for inflammasome components over disease progression [20].

In CL, we observed a positive correlation between macrophage subsets (CD68+, CD163+) and inflammasome markers (IL‐1β, IL‐18, Caspase‐1), suggesting that inflammasome activation occurs in both inflammatory and anti‐inflammatory macrophage populations. In MCL, CD163^+^ correlated positively with IL‐1β^+^ cells in the skin; however, it correlated negatively with IL‐1β^+^ and Caspase‐1^+^ cells in mucosal lesions, as well as showing a negative correlation between the density of CD68^+^ and IL‐1β^+^ cells. These findings suggest a prominent role for inflammasome activation in chronic cutaneous lesions of CL and MCL, associated with macrophage infiltration and high parasite loads. Notably, inflammasome markers correlated positively with parasite density in skin and negatively in mucosa. Supporting this, studies in *L. (L.) mexicana* cutaneous infections report IL‐1β distribution varying by clinical form and parasite burden [42]. As a cross‐sectional design, it does not allow causal inference; therefore, although a reduction in parasite burden mediated by inflammasome activation is suggested, it cannot be established whether inflammasome activation occurs as a cause or consequence of the amount of parasites.

Since this study was retrospective, using samples collected at varying stages of disease progression, without standardised sampling procedures, and from different body sites, these factors may also have influenced the results. Therefore, controlled prospective studies are needed to better elucidate the suggested mechanisms.

Based on our findings, we propose a model in which, in CL, the high parasite burden and intense macrophage infiltration promote NLRP3 inflammasome activation in both M1 and M2 subpopulations, increasing caspase‐1 activity and IL‐1β/IL‐18 secretion. In M1 macrophages, such activation induces nitric oxide production, parasite control, and pyroptosis, whereas in M2 macrophages, IL‐10 secretion modulates inflammation. In MCL, the low parasite burden is associated with reduced inflammasome activation, suggesting suppression of the innate immune response without preventing chronic inflammation sustained by macrophages and Th1 lymphocytes, ultimately leading to greater tissue damage. Future studies could be conducted to contribute to our findings using M1/M2 macrophages isolated from infected patients, with and without genetic or pharmacological inhibition of the inflammasome, and murine models deficient in these components, assessing lesion development, parasite burden, polarisation, and inflammatory mediators.

Overall, our findings indicate that inflammasome components play a crucial role in the chronic, non‐specific inflammatory response associated with CL and MCL skin lesions, probably contributing to reducing the high tissue parasitism. In contrast, in mucosal lesions, where the cellular immune response is more chronically defined and tissue parasitism is rare, the involvement of the inflammasome appears to be less significant. In conclusion, our preliminary study demonstrates a differential involvement of the NLRP3 inflammasome and macrophage subpopulations in CL and MCL, influencing the in situ inflammatory response and tissue parasitism.

## Author Contributions

All authors contributed significantly to the development of this work. Caroline Heleno Chagas de Carvalho participated in the study's conception and design, data collection, data analysis, interpretation of results, and manuscript writing. Gabriela Venicia Araujo Flores contributed to laboratory bench work, data analysis, interpretation of results, and manuscript writing. Carmen Maria Sandoval Pacheco contributed to laboratory bench work, language review, and final text editing. Vania Lucia Ribeiro da Matta contributed to the molecular diagnosis and species identification. Carolina Esteves de Morais contributed to the histopathological analysis of the biopsy blocks. Ricardo Romiti contributed to the critical review, language editing, and final revision of the manuscript. Walter Belda Júnior contributed to the study's conception and design, and the critical review of the manuscript. Márcia Dalastra Laurenti participated in the study's conception and design, data analysis, interpretation of results, writing, and critical review of the manuscript. All authors approved the final version of the article and are responsible for the content presented.

## Ethics Statement

This study was approved by the Ethics Committee for Research Project Analysis (CAPPesq) of the Hospital das Clínicas, Faculty of Medicine, University of São Paulo (HC‐FMUSP), Brazil, under approval number 5.207.836. Confidentiality and anonymization of patient data were ensured.

## Conflicts of Interest

The authors declare no conflicts of interest.

## Peer Review

The peer review history for this article is available at https://www.webofscience.com/api/gateway/wos/peer‐review/10.1111/pim.70026.

## Supporting information


**Supporting 1.** PCR‐RFLP Hsp70 (A): Digestion of amplicons from paraffin‐embedded biopsies with *Hae* III. (A), (B), (C), (D), and (E) correspond to skin samples from cutaneous leishmaniasis. **PCR‐RFLP ITS1 (B):** Digestion of amplicons from paraffin‐embedded biopsies using *Hae* III (**A**) and (**C**) correspond to skin samples from cutaneous leishmaniasis, (**B**) mucosal tissue, and (**D**) skin from mucocutaneous leishmaniasis. Positive controls: Lb = *L. (V.) braziliensis* (MHOM/BR/1995/M15280); La = *L. (L.) amazonensis* (MHOM/BR/1973/M2269); Ls = *L. (V.) shawi* (MCEB/BR/1984/M8408); Li = *L. (L.) infantum* (MHOM/BR/1974/PP75); PM: molecular weight of 25 base pairs.

**Supporting 2.** Illustrative image of the immunohistochemical reaction in a histological section of a skin biopsy from patients with cutaneous leishmaniasis, showing (A) CD68‐positive cells, (B) CD163‐positive cells, (C) iNOS‐positive cells, and (D) IL‐10‐positive cells in brown colour. DAB staining, objective 40×.

**Supporting 3.** Illustrative image of the immunohistochemical reaction in a histological section of a healthy skin biopsy, showing (A) CD68‐positive cells, (B) CD163‐positive cells, (C) iNOS‐positive cells, and (D) IL‐10‐positive cells in brown colour.

**Supporting 4.** Illustrative image of the immunohistochemical reaction in a histological section of a healthy skin biopsy, showing (A) IL‐1𝛃‐positive cells, (B) IL‐18‐positive cells, and (C) caspase‐1‐positive cells in brown colour.

## Data Availability

The data that support the findings of this study are available from the corresponding author upon reasonable request.
