# Supplementary figures and images for: In Situ Evaluation of Macrophage Populations and Inflammasome Components in Cutaneous and Mucocutaneous Leishmaniasis
Source: Parasite Immunol. 2025 Sep 14;47(9):e70026. doi: 10.1111/pim.70026 (PMC12434389; doi:10.1111/pim.70026)

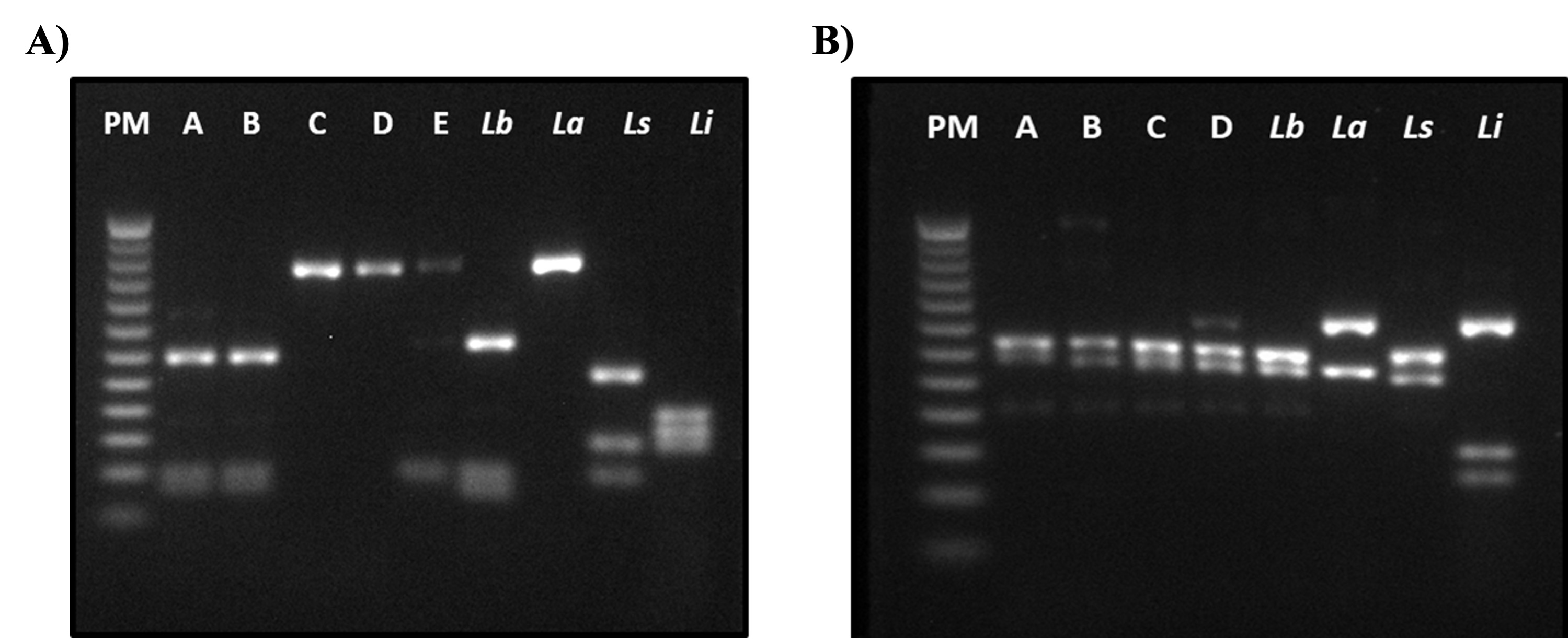

Supplement: Supplementary file 1 — Supporting 1. PCR‐RFLP Hsp70 (A): Digestion of amplicons from paraffin‐embedded biopsies with Hae III. (A), (B), (C), (D), and (E) correspond to skin samples from cutaneous leishmaniasis. PCR‐RFLP ITS1 (B): Digestion of amplicons from paraffin‐embedded biopsies using Hae III (A) and (C) correspond to skin samples from cutaneous leishmaniasis, (B) mucosal tissue, and (D) skin from mucocutaneous leishmaniasis. Positive controls: Lb = L. (V.) braziliensis (MHOM/BR/1995/M15280); La = L. (L.) amazonensis (MHOM/BR/1973/M2269); Ls = L. (V.) shawi (MCEB/BR/1984/M8408); Li = L. (L.) infantum (MHOM/BR/1974/PP75); PM: molecular weight of 25 base pairs. [file PIM-47-e70026-s004.zip › pim70026-sup-0003-Supinfo3@Supplementary 1.jpg]

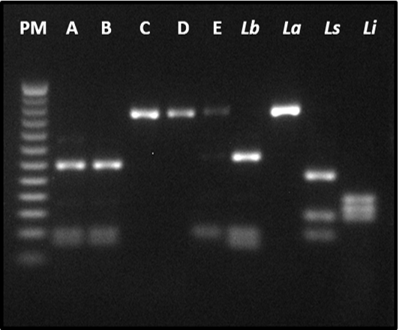

Supplement: Supplementary file 1 — Supporting 1. PCR‐RFLP Hsp70 (A): Digestion of amplicons from paraffin‐embedded biopsies with Hae III. (A), (B), (C), (D), and (E) correspond to skin samples from cutaneous leishmaniasis. PCR‐RFLP ITS1 (B): Digestion of amplicons from paraffin‐embedded biopsies using Hae III (A) and (C) correspond to skin samples from cutaneous leishmaniasis, (B) mucosal tissue, and (D) skin from mucocutaneous leishmaniasis. Positive controls: Lb = L. (V.) braziliensis (MHOM/BR/1995/M15280); La = L. (L.) amazonensis (MHOM/BR/1973/M2269); Ls = L. (V.) shawi (MCEB/BR/1984/M8408); Li = L. (L.) infantum (MHOM/BR/1974/PP75); PM: molecular weight of 25 base pairs. [file PIM-47-e70026-s004.zip › pim70026-sup-0001-Supinfo1@Supplementary 1 a.png]

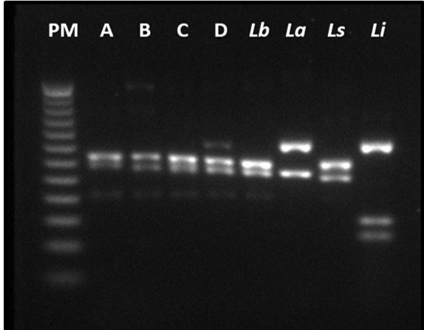

Supplement: Supplementary file 1 — Supporting 1. PCR‐RFLP Hsp70 (A): Digestion of amplicons from paraffin‐embedded biopsies with Hae III. (A), (B), (C), (D), and (E) correspond to skin samples from cutaneous leishmaniasis. PCR‐RFLP ITS1 (B): Digestion of amplicons from paraffin‐embedded biopsies using Hae III (A) and (C) correspond to skin samples from cutaneous leishmaniasis, (B) mucosal tissue, and (D) skin from mucocutaneous leishmaniasis. Positive controls: Lb = L. (V.) braziliensis (MHOM/BR/1995/M15280); La = L. (L.) amazonensis (MHOM/BR/1973/M2269); Ls = L. (V.) shawi (MCEB/BR/1984/M8408); Li = L. (L.) infantum (MHOM/BR/1974/PP75); PM: molecular weight of 25 base pairs. [file PIM-47-e70026-s004.zip › pim70026-sup-0002-Supinfo2@Supplementary 1 b.png]

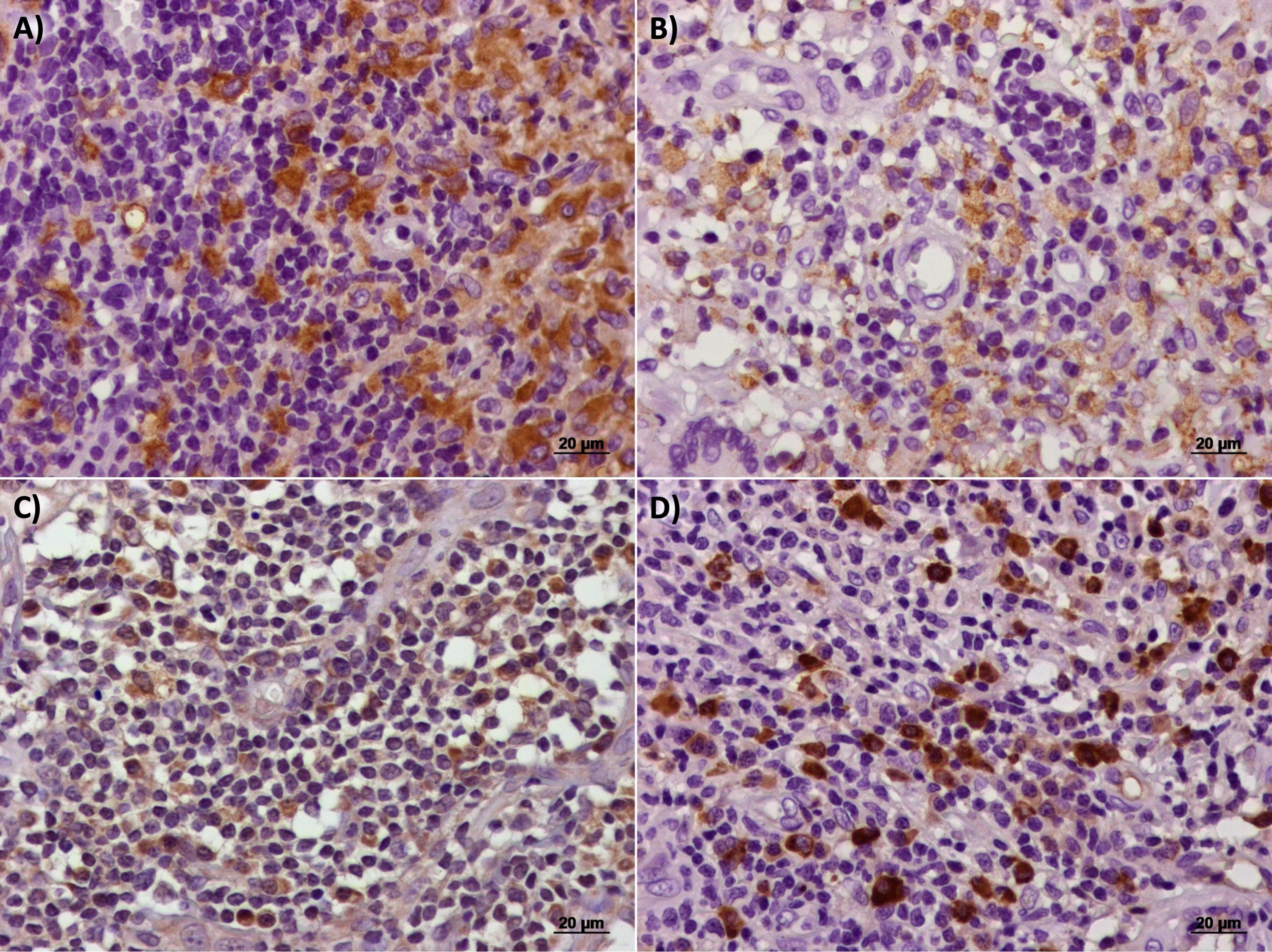

Supplement: Supplementary file 2 — Supporting 2. Illustrative image of the immunohistochemical reaction in a histological section of a skin biopsy from patients with cutaneous leishmaniasis, showing (A) CD68‐positive cells, (B) CD163‐positive cells, (C) iNOS‐positive cells, and (D) IL‐10‐positive cells in brown colour. DAB staining, objective 40×. [file PIM-47-e70026-s003.zip › pim70026-sup-0005-Supinfo5@Supplementary 2.jpg]

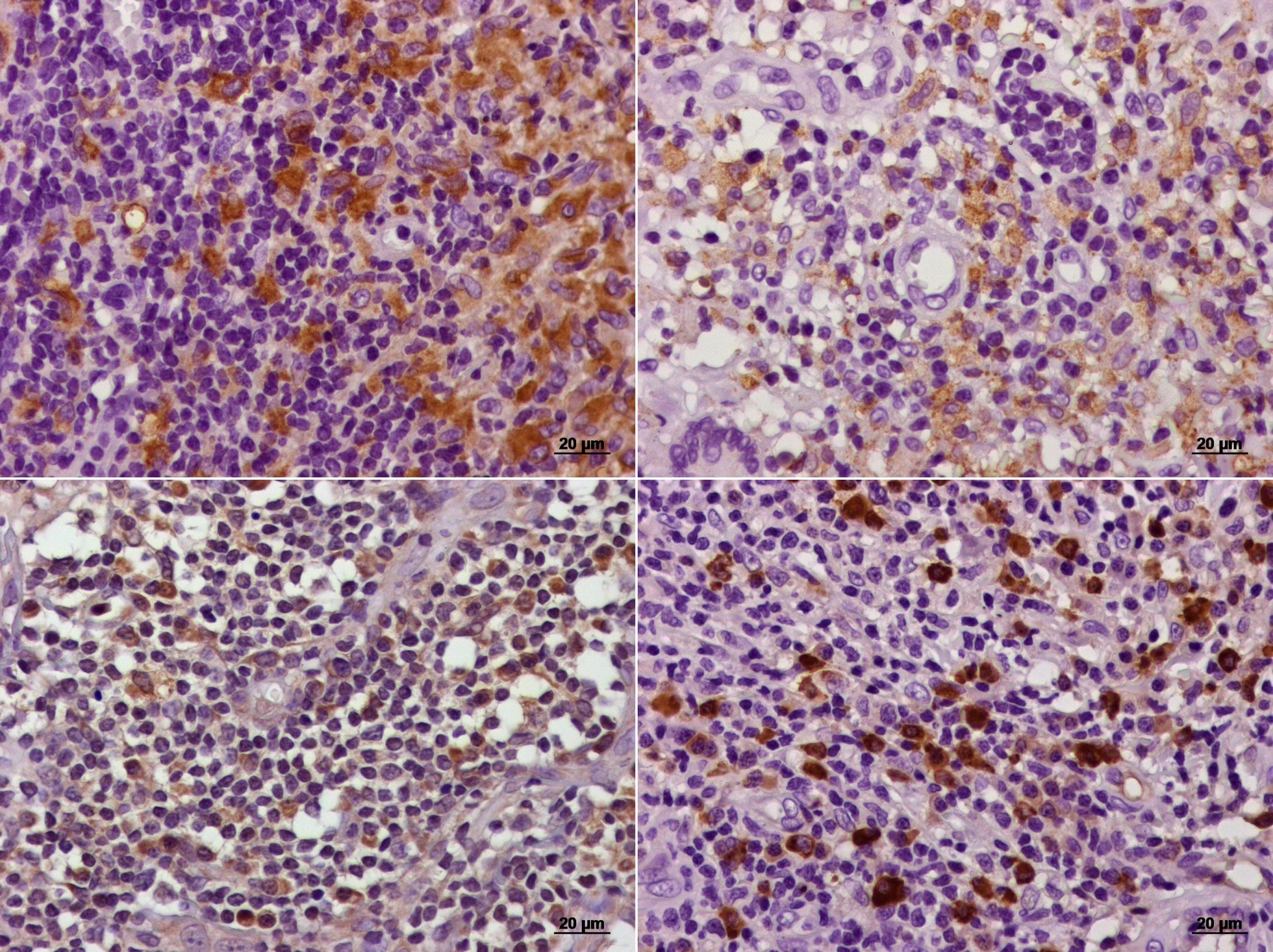

Supplement: Supplementary file 2 — Supporting 2. Illustrative image of the immunohistochemical reaction in a histological section of a skin biopsy from patients with cutaneous leishmaniasis, showing (A) CD68‐positive cells, (B) CD163‐positive cells, (C) iNOS‐positive cells, and (D) IL‐10‐positive cells in brown colour. DAB staining, objective 40×. [file PIM-47-e70026-s003.zip › pim70026-sup-0004-Supinfo4@Supplementary 2 abcd.jpg]

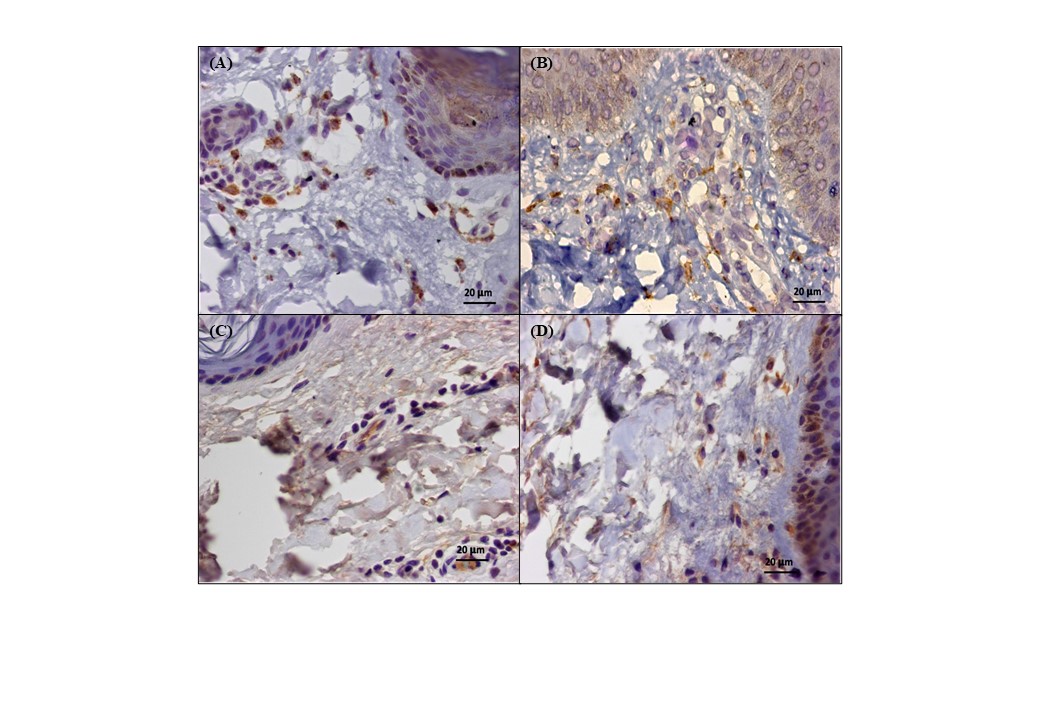

Supplement: Supplementary file 3 — Supporting 3. Illustrative image of the immunohistochemical reaction in a histological section of a healthy skin biopsy, showing (A) CD68‐positive cells, (B) CD163‐positive cells, (C) iNOS‐positive cells, and (D) IL‐10‐positive cells in brown colour. [file PIM-47-e70026-s001.jpg]

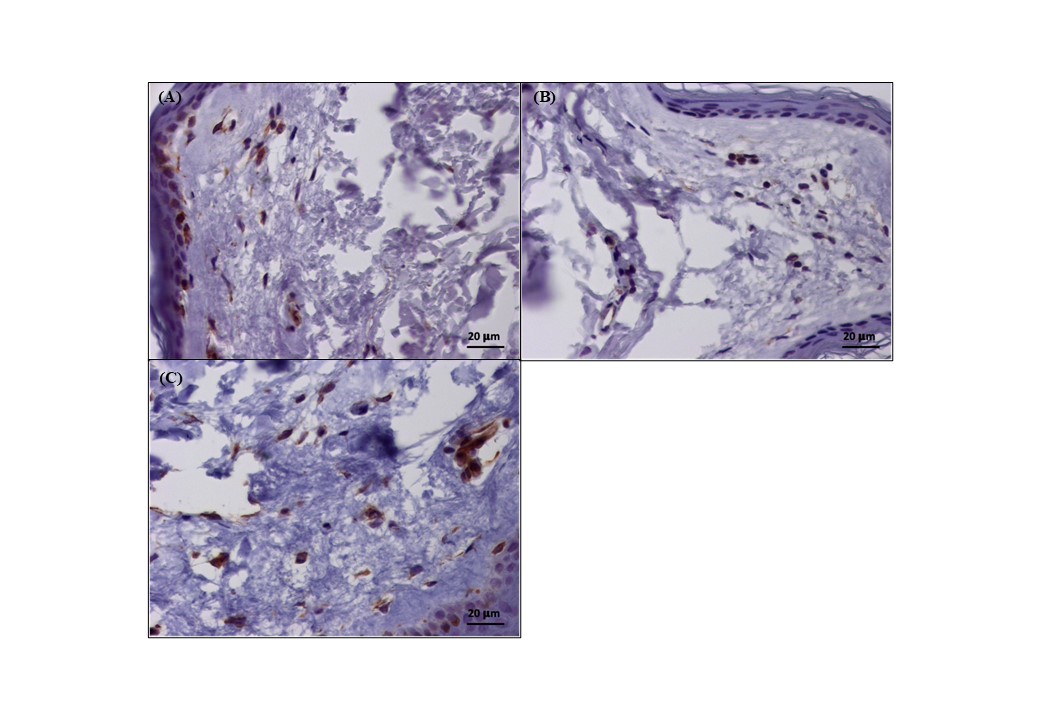

Supplement: Supplementary file 4 — Supporting 4. Illustrative image of the immunohistochemical reaction in a histological section of a healthy skin biopsy, showing (A) IL‐1𝛃‐positive cells, (B) IL‐18‐positive cells, and (C) caspase‐1‐positive cells in brown colour. [file PIM-47-e70026-s002.jpg]
